# SLC16A7 and WDR38 form a mitochondrial-centric axis driving lung cancer through metabolic reprogramming and REDOX adaptation

**DOI:** 10.1515/jtim-2025-0056

**Published:** 2025-12-29

**Authors:** Jungang Zhao, Yuge Zhao, Jun Tang, Zhiqiang Wu, Wen Li, Yubo Yan

**Affiliations:** Shengjing Hospital Affiliated to China Medical University, Shenyang, Liaoning Province, China; Wright State University, Dayton, OH, USA; Yongzhou Zhong Gu Biotechnology Co., Ltd, Yongzhou, Hunan Province, China; Harbin Medical University Cancer Hospital, Harbin, Heilongjiang Province, China

## Introduction

Lung cancer is the leading cause of cancer-related mortality globally (WHO, 2022).^[1]^ Current protein biomarkers for lung cancer suffer from limited sensitivity (50%–70%) and specificity (65%–80%).^[2,3]^ Recent studies highlight the potential roles of solute carrier family 16 member 7 (SLC16A7) and WD repeat domain 38 (WDR38) in lung cancer pathogenesis.^[4, 5, 6]^ SLC16A7, a monocarboxylate transporter, facilitates lactate efflux from glycolytic tumor cells, acidifying the extracellular matrix to drive invasion and immune evasion. Its overexpression in squamous cell carcinoma is associated with poor prognosis and resistance to anti-angiogenic therapies. WDR38, a scaffold protein involved in homologous recombination repair, maintains genomic stability by recruiting BRCA1 to DNA double-strand break sites. Somatic mutations in WDR38 (observed in 1.2% of NSCLC cases) disrupt this interaction, conferring synthetic lethality with PARP inhibitors, and WDR38 expression levels are closely related to the REDOX state of the cells. Lung cancer cells protect themselves from oxidative stress by enhancing the expression and activity of antioxidant enzymes and reducing the production of reactive oxygen species (ROS).^[7]^ In this study, we systematically investigated into the roles of SLC16A7 and WDR38 in maintaining the intracellular REDOX balance and promoting the survival and spread of lung cancer cells.

## Method

### Integrated normalization and batch correction for cross-dataset PCA in lung cancer Transcriptomics

Two lung cancer transcriptomic datasets (GSE229301: 3 tumor-adjacent *vs*. 3 tumor samples; GSE136043: 5 non-cancerous *vs*. 5 tumor samples) were retrieved from GEO using the GPL13497 Agilent-026652 Whole Human Genome Microarray platform.

### Differential expression analysis of integrated lung cancer transcriptomic datasets

Differentially expressed genes (DEGs) were identified from the merged GSE229301 and GSE136043 datasets using R packages limma, dplyr, pheatmap, and ggplot2.

### Functional enrichment profiling of lung cancer DEGs via GO and KEGG pathways

Gene Ontology (GO) and Kyoto Encyclopedia of Genes and Genomes (KEGG) enrichment analyses were performed on lung cancer DEGs using R packages clusterProfiler, org.Hs.eg.db, enrichplot, ggplot2, ComplexHeatmap, and RColorBrewer.

### LASSO regression for feature selection in lung cancer transcriptomics

Least Absolute Shrinkage and Selection Operator (LASSO) regression was applied to the lung cancer DEGs using the glmnet package in R, with reproducibility ensured by setting set.seed(12345).

### Random forest modeling for feature prioritization in lung cancer Transcriptomics

Random forest analysis was performed on lung cancer DEGs using R packages randomForest, ggplot2, ggpubr, viridis, and dplyr. Model reproducibility was ensured with set.seed(12345).

### Integrative analysis of overlapping biomarkers and mutation landscapes in lung Cancer

Overlapping genes between LASSO and random forest (RF) models were identified using the online Venn diagram tool (http://bioinformatics.psb.ugent.be/webtools/Venn/). Mutation profiles of prioritized genes were analyzed using level 4 Simple Nucleotide Variation data from TCGA lung adenocarcinoma (LUAD) and squamous cell carcinoma (LUSC) cohorts (*n* = 5080 LUAD, *n* = 485 LUSC), downloaded *via* the GDC portal (https://portal.gdc.cancer.gov/) and preprocessed with MuTect2 (DOI:10.1038/nature08822).

### Integrated analysis of mitochondrial protein expression and interaction networks in lung cancer

Mitochondrial proteins were retrieved from the MitoCarta database and integrated with merged transcriptomic datasets (GSE229301 and GSE136043) from lung cancer samples. Differential expression analysis was performed using the limma package in R. Protein-protein interaction (PPI) networks for upregulated genes were constructed *via* the STRING database, with a confidence score cutoff of 0.15 to prioritize functional associations.

## Results

### Batch effect correction unifies dataset distributions in lung cancer transcriptomic PCA

PCA revealed distinct clustering patterns between datasets before and after batch correction (Supplementary Figure S1). The elimination of dataset-specific spatial bias confirmed successful batch effect mitigation, as evidenced by interspersed GSE136043 and GSE229301 samples across principal components. These results demonstrate that sva-based correction enhances inter-dataset compatibility, enabling robust integrative analysis of heterogeneous lung cancer cohorts.

### Robust Transcriptional Dysregulation in lung cancer Reveals 942 biomarker candidates

Differential analysis identified 942 DEGs (592 downregulated, 350 upregulated) in tumor tissues *versus* controls (Supplementary Figure S2).

### Lung cancer DEGs implicate developmental and signaling Pathways in tumor pathogenesis

GO and KEGG analyses revealed distinct functional themes among 942 DEGs (Supplementary Figure S3).

### LASSO Regression Prioritizes SLC16A7, DDAH2, and WDR38 as Core Lung Cancer Biomarkers

LASSO regression identified three genes (SLC16A7, DDAH_2_, WDR38) with non-zero coefficients at the optimal λ (0.2626) (Supplementary Figure S4).

### Random forest model identifies 30 mechanistically relevant genes in lung Cancer

The random forest model achieved stable classification (OOB error rate ≤0.06) after 250 trees, with error rates plateauing beyond this threshold (Supplementary Figure S5A). The error rate trajectory (orange dashed line) confirmed robust model performance below the 0.1 threshold. Feature importance analysis (Supplementary Figure S5B) prioritized 30 genes (Gini index: 0.04–0.12), including top-ranked WDR38 (0.12) and EFNB3 (0.10). High-scoring genes encompassed ion transporters (SLC16A7, CLEC5A), signaling regulators (FZD8, SEMA3D, JAKMIP2), and extracellular matrix modulators (WISP2, RETNLB). Horizontal bars in displayed a viridis color gradient (purple: low importance, yellow: high), with gene labels ordered by descending Gini scores. These results nominate 30 genes as central to lung cancer pathogenesis, with implications for therapeutic targeting.

### Overlapping biomarkers DDAH2, SLC16A7, and WDR38 Exhibit recurrent mutations in lung cancer

Venn analysis identified three overlapping genes (DDAH_2_, SLC16A7, WDR38) between LASSO and RF models (Supplementary Figure S6A). Mutation profiling revealed WDR38 mutations in 0.98% (5/508) of LUAD and 0.82% (4/485) of LUSC cases (Supplementary Figure S6B), predominantly missense mutations (blue circles) distributed across the protein. SLC16A7 (Supplementary Figure S6C) exhibited sparse missense mutations (*e.g*., p. N58L, p. S125I) in LUAD (1.2% mutation rate), with no LUSC variants detected. DDAH_2_ (Supplementary Figure S6D) displayed a nonsense mutation hotspot (p. W356*, green circle) in both LUAD (0.2%) and LUSC (0.6%), alongside missense variants. Gray backgrounds denote conserved protein domains (*e.g*., amidinotransferase domain in DDAH_2_). These results nominate DDAH_2_, SLC16A7, and WDR38 as recurrently mutated, functionally relevant biomarkers in lung cancer.

### Mitochondrial protein dysregulation in lung cancer reveals key upregulated networks and biomarkers

**Figure 1:**
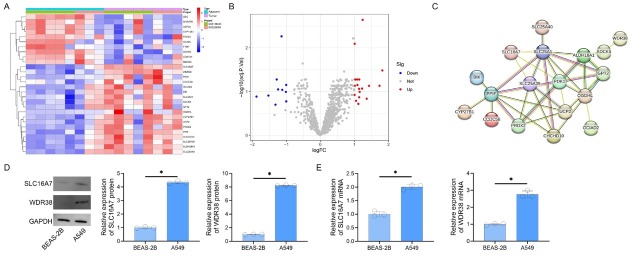
Differential Expression and Interaction. Networks of Mitochondrial Proteins in Lung Cancer. A. Heatmap of log2(fold change)-scaled expression for 28 differentially expressed mitochondrial proteins. Red and blue denote up- and downregulation, respectively. B. Volcano plot illustrating differential expression (|log2FC| > 1, adjusted *P* < 0.05). Red/blue dots represent significant genes; gray indicates non-significant candidates. C. Protein-protein interaction network of 18 upregulated mitochondrial genes, with edges weighted by interaction confidence (STRING score ≥ 0.15). D. Western blot analysis of SLC16A7 and WDR38 protein expression (GAPDH loading control). E. RT-PCR quantification of SLC16A7 and WDR38 mRNA levels (^*^P < 0.05 *vs*. BEAS-2B).

Clustered heatmap visualization demonstrated consistent overexpression of SLC16A7 and WDR38 across tumor samples compared to normal tissues (Figure 1A). Volcano plot analysis confirmed their significant differential expression (Figure 1B). Bioinformatic analysis of merged lung cancer datasets identified 28 mitochondrial-associated DEGs, including SLC16A7 and WDR38 as core interactors within the PPI network (Figure 1C). STRING network analysis revealed SLC16A7 and WDR38 as central nodes, directly interacting with 12 and 9 mitochondrial proteins, respectively, predominantly enriched in oxidative phosphorylation and tricarboxylic acid cycle regulation. Experimental validation *via* western blot and RT-PCR confirmed elevated expression of SLC16A7 and WDR38 proteins and transcripts in A549 *versus* BEAS-2B cells (Figures 1D, 1E), supporting their functional centrality in mitochondrial dysregulation.

## Discussion

The metabolic reprogramming and REDOX adaptation mechanisms of tumor cells is a key path to decoding the molecular foundation of tumor occurrence and development. As a key transmembrane transporter protein, SLC16A7 is significantly up-regulated in lung cancer cells and is highly positively correlated with tumor invasivity and metastasis potential. When the expression of SLC16A7 is inhibited, the intracellular lactic acid concentration rises sharply, subsequently triggering the activation of the AMPK energy sensing pathway. The interaction between SLC16A7 and HIF-1α enables tumor cells to obtain energy by enhancing the glycolytic pathway in the case of insufficient oxygen supply, while promoting angiogenesis to improve the supply of oxygen and nutrients. SLC16A7 is also deeply involved in shaping the tumor immune microenvironment and has a complex interaction pattern with infiltrating immune cells such as T lymphocytes and natural killer cells, leading to changes that are not conducive to their functional performance, evading the surveillance and clearance of the body’s immune system.

WDR38, with its unique WD40 domain, plays an irreplaceable role in maintaining mitochondrial intimal homeostasis and reshaping energy metabolism. In lung cancer cell lines, WDR38 can specifically bind to multicomponent protein complexes within mitochondria, precisely regulate the activities of each component of the electron transport chain, and regulate the mitochondrial autophagy process.

In the synergistic mechanism of the mitochondrial central protein axis in the metabolic remodeling of lung cancer, SLC16A7 and WDR38 form a tight functional coupling in this axis, and the synergistic effect of the two significantly regulate the ATP level and effectively inhibit the ROS level, providing a solid metabolic guarantee for the continuous proliferation and survival of tumor cells in a complex microenvironment. By targeting and intervening in this protein axis and disrupting its fine regulation of cellular metabolism and REDOX states, the sensitivity of lung cancer cells to chemotherapy drugs and radiotherapy may be reactivated.^[8,9]^

## Conclusion

This study presents the molecular mechanisms of SLC16A7 and WDR38 in lung cancer cells development involving metabolic reprogramming and REDOX adaptation. Through interaction and synergy, they jointly promote the growth, survival and development of drug resistance of lung cancer cells.

## Supplementary Information

Supplementary materials are only available at the official site of the journal (www.intern-med.com).

## Supplementary Material

Supplementary Material Details
