# Supplementary material for: SLC16A7 and WDR38 form a mitochondrial-centric axis driving lung cancer through metabolic reprogramming and REDOX adaptation
Source: J Transl Int Med. 2025 Dec 29;14(2):315–8. doi: 10.1515/jtim-2025-0056 (PMC13110461; doi:10.1515/jtim-2025-0056)
Supplement: Supplementary file 1 — Supplementary Material Details [file jtim-2025-0056_sm.pdf]

Supplementary materials

SLC16A7 and WDR38 Form a Mitochondrial-Centric Axis Driving Lung Cancer through Metabolic Reprogramming and Redox Adaptation

Supplementary material

Detailed Method

Integrated Normalization and Batch Correction for Cross-Dataset PCA in Lung Cancer Transcriptomics

Two lung cancer transcriptomic datasets (GSE229301: 3 tumor-adjacent *vs.* 3 tumor samples; GSE136043: 5 non-cancerous *vs.* 5 tumor samples) were retrieved from GEO using the GPL13497 Agilent-026652 Whole Human Genome Microarray platform. Raw data matrices were extracted using Perl scripts, followed by inter-dataset normalization with the limma package in R to mitigate technical variability. Batch effects between datasets were corrected using the sva package's ComBat algorithm to enable integration. Principal Component Analysis (PCA) was performed using stats (v3.6.0) after z-score transformation of expression profiles, with dimensionality reduction executed via the prcomp function to generate PCA coordinates. This pipeline ensured comparability of cross-platform datasets for downstream analyses.

Differential Expression Analysis of Integrated Lung Cancer Transcriptomic Datasets

Differentially expressed genes (DEGs) were identified from the merged GSE229301 and GSE136043 datasets using R packages limma, dplyr, pheatmap, and ggplot2. Raw expression matrices were subjected to linear modeling *via* limma to estimate log-fold changes (logFC) and p-values, followed by empirical Bayes moderation for variance stabilization. DEGs were filtered using thresholds of $|logFC| > 1$ and adjusted *P*-value < 0.05. The dplyr package facilitated data subsetting and annotation, while pheatmap generated a hierarchical clustering heatmap (z-score normalized expression values, Euclidean distance, complete linkage). ggplot2 produced a volcano plot to visualize logFC against -log10(*P*-value). This workflow ensured rigorous identification and visualization of transcriptional perturbations in lung cancer.

Functional Enrichment Profiling of Lung Cancer DEGs *via* GO and KEGG Pathways

Gene Ontology (GO) and Kyoto Encyclopedia of Genes and Genomes (KEGG) enrichment analyses were performed on lung cancer DEGs using R packages clusterProfiler, org.Hs.eg.db, enrichplot, ggplot2, ComplexHeatmap, and RColorBrewer. Gene identifiers were annotated *via* org.Hs.eg.db, followed by GO analysis (biological processes, molecular functions, cellular components) and KEGG pathway mapping. Enrichment significance thresholds were set at raw *P*-value ≤ 0.05 and adjusted p-value (Benjamini-Hochberg) ≤ 0.05. Bar plots and dimension-reduced scatterplots (elliptical clustering) were generated using ggplot2 and enrichplot to visualize term/pathway enrichment. ComplexHeatmap and RColorBrewer optimized color gradients for adjusted *P*-values (red: low significance, blue: high) and gene counts (bubble/ellipse size). This pipeline ensured systematic identification of functionally coherent biological themes.

LASSO Regression for Feature Selection in Lung Cancer Transcriptomics

Least Absolute Shrinkage and Selection Operator (LASSO) regression was applied to the lung cancer DEGs using the glmnet package in R, with reproducibility ensured by setting set.seed(12345). The penalized regression model minimized the binomial deviance while imposing an L1-norm penalty to shrink irrelevant gene coefficients to zero. Ten-fold cross-validation was performed to identify the optimal regularization parameter (λ), balancing model simplicity and predictive accuracy. The cross-validation curve and coefficient shrinkage paths were visualized using glmnet's built-in plotting functions. Gene importance rankings were derived from absolute coefficient magnitudes at the optimal λ. This approach prioritized genes with robust associations to lung cancer phenotypes while mitigating overfitting.

Random Forest Modeling for Feature Prioritization in Lung Cancer Transcriptomics

Random forest analysis was performed on lung cancer DEGs using R packages randomForest, ggplot2, ggpubr, viridis, and dplyr. Model reproducibility was ensured with set.seed(12345). A classification forest with 1000 decision trees was trained, and out-of-bag (OOB) error rates were monitored across

incremental tree counts (0–1000) to assess convergence stability. Ten-fold cross-validation optimized hyperparameters. Feature importance was quantified via mean decrease in Gini index, with genes scoring >0.04 retained as high-priority candidates. ggplot2 and ggpubr generated line plots (error rates *vs*. tree count) and horizontal bar plots (importance rankings), while viridis provided color gradients to enhance visualization clarity. Data wrangling and annotation were streamlined using dplyr.

Integrative Analysis of Overlapping Biomarkers and Mutation Landscapes in Lung Cancer

Overlapping genes between LASSO and random forest (RF) models were identified using the online Venn diagram tool (http://bioinformatics.psb.ugent.be/webtools/Venn/). Mutation profiles of prioritized genes were analyzed using level 4 Simple Nucleotide Variation data from TCGA lung adenocarcinoma (LUAD) and squamous cell carcinoma (LUSC) cohorts (n = 5080 LUAD, n = 485 LUSC), downloaded via the GDC portal (https://portal.gdc.cancer.gov/) and preprocessed with MuTect2 (DOI:10.1038/nature08822). Mutation annotation format (MAF) files were integrated using the maftools package (v2.2.10) in R to calculate mutation frequencies, classify variant types (missense, nonsense), and map mutations to protein structural domains. Mutation landscapes were visualized via the http://sangerbox.com/platform, with protein domain annotations extracted from maftools to contextualize mutation hotspots.

Integrated Analysis of Mitochondrial Protein Expression and Interaction Networks in Lung Cancer

Mitochondrial proteins were retrieved from the MitoCarta database and integrated with merged transcriptomic datasets (GSE229301 and GSE136043) from lung cancer samples. Differential expression analysis was performed using the limma package in R, applying thresholds of |log2(fold change)| > 1 and adjusted *P*-value < 0.05 to identify significant genes. Downstream visualization, including hierarchical clustering heatmaps (log2FC-scaled) and volcano plots, was generated using ggplot2 and pheatmap. Protein-protein interaction (PPI) networks for upregulated genes were constructed via the STRING database, with a confidence score cutoff of 0.15 to prioritize functional associations.

Detailed Results

Batch Effect Correction Unifies Dataset Distributions in Lung Cancer Transcriptomic PCA

PCA revealed distinct clustering patterns between datasets before and after batch correction (Supplementary Figure S1). In the pre-correction analysis (Supplementary Figure S1A), GSE136043 samples (blue squares) occupied the upper-right quadrant (PC1: 37.8%, PC2: 18%), while GSE229301 samples (yellow triangles) clustered centrally with leftward dispersion, forming partially overlapping yet separable groups within an elliptical boundary. Post-correction (Supplementary Figure S1B), both datasets exhibited uniform dispersion across quadrants, with merged clusters encapsulated by a single elliptical region (PC1: 31.6%, PC2: 20.8%). The elimination of dataset-specific spatial bias confirmed successful batch effect mitigation, as evidenced by interspersed GSE136043 and GSE229301 samples across principal components. These results demonstrate that sva-based correction enhances inter-dataset compatibility, enabling robust integrative analysis of heterogeneous lung cancer cohorts.

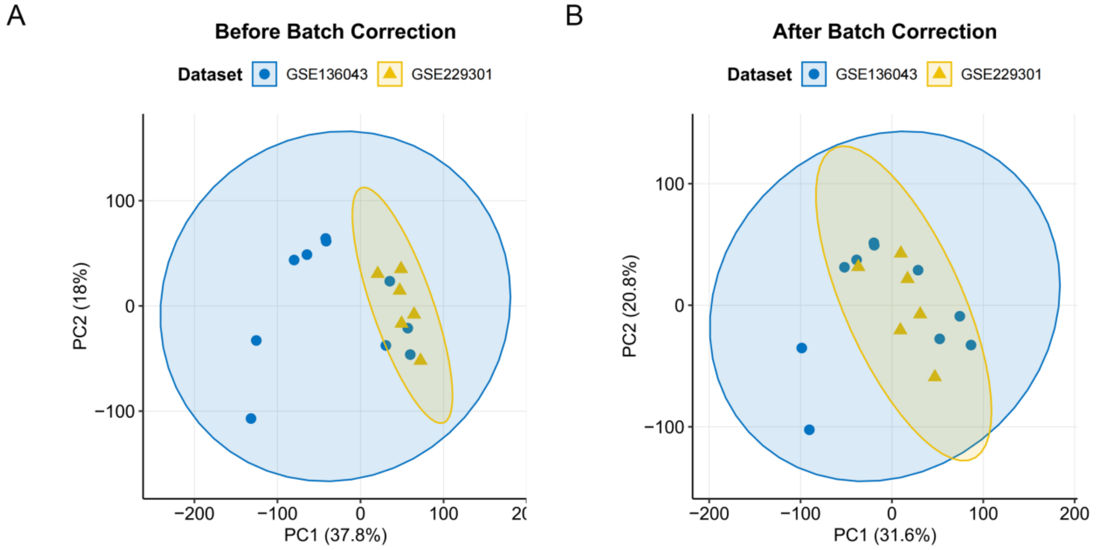

Supplementary Figure S1. Spatial Integration of Lung Cancer Transcriptomic Datasets Before and After Batch Correction. A. Before Batch Correction: Discrete clustering of GSE136043 (blue squares) and GSE229301 (yellow triangles) datasets with partial overlap (elliptical boundary, PC2: 18%). B. After Batch Correction: Unified distribution of both datasets within a consolidated elliptical region (PC2: 20.8%).

Robust Transcriptional Dysregulation in Lung Cancer Reveals 942 Biomarker Candidates

Differential analysis identified 942 DEGs (592 downregulated, 350 upregulated) in tumor tissues *versus* controls (Supplementary Figure S2). The heatmap (Supplementary Figure S2A) demonstrated clear segregation between tumor and adjacent/non-cancer samples, with hierarchical clustering revealing two dominant gene clusters: a red-enriched group (upregulated in tumors) and a blue-enriched group (downregulated). Intense color gradients at the heatmap margins indicated genes with extreme expression shifts (logFC > 1 or < -1). The volcano plot (Supplementary Figure S2B) highlighted 592 significantly downregulated (blue, left quadrant) and 350 upregulated (red, right quadrant) genes, with -log10(*P*-value) exceeding 1.3 ($P < 0.05$) for all DEGs. Non-significant genes (gray) clustered near the origin, confirming stringent filtering. These findings underscore widespread transcriptional dysregulation in lung cancer, dominated by tumor-suppressive gene downregulation.

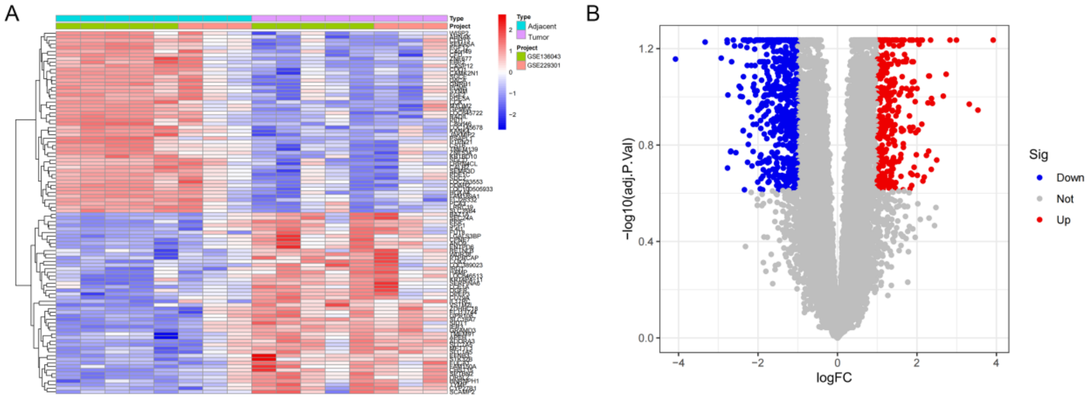

Supplementary Figure S2. Transcriptional Landscape of Lung Cancer Tissues Highlights Widespread Differential Expression. A. Heatmap: Hierarchical clustering of 942 DEGs (rows) and samples (columns). Red indicates upregulation, blue indicates downregulation in tumors (z-score scaled). B. Volcano Plot: Distribution of DEGs by logFC (x-axis) and -log10(*P*-value) (y-axis). Red: upregulated (350 genes), blue: downregulated (592 genes), gray: non-significant.

Lung Cancer DEGs Implicate Developmental and Signaling Pathways in Tumor Pathogenesis

GO and KEGG analyses revealed distinct functional themes among 942 DEGs (Supplementary Figure S3). GO biological processes (Supplementary Figure S3A) highlighted significant enrichment in "renal system development" (P = 0.0000025, 40 genes) and "muscle system process" (P = 0.000015, 35 genes), with bar lengths correlating to gene counts. The dimension-reduced GO scatterplot (Supplementary Figure S3B) clustered terms like "cell adhesion" and "extracellular matrix structure" into ellipsoid groups, reflecting functional synergy (adjusted $P < 0.001$). KEGG pathways (Supplementary Figure S3C) identified "Calcium signaling pathway" (P = 0.0005, 15 genes) and "ECM-receptor interaction" (P = 0.001, 12 genes) as top dysregulated pathways. The KEGG scatterplot (Supplementary Figure S3D) further grouped pathways such as "Viral myocarditis" and "Cytoskeleton in muscle cells" into elliptical clusters, emphasizing shared regulatory mechanisms. These findings link lung cancer DEGs to developmental dysregulation and extracellular signaling aberrations,

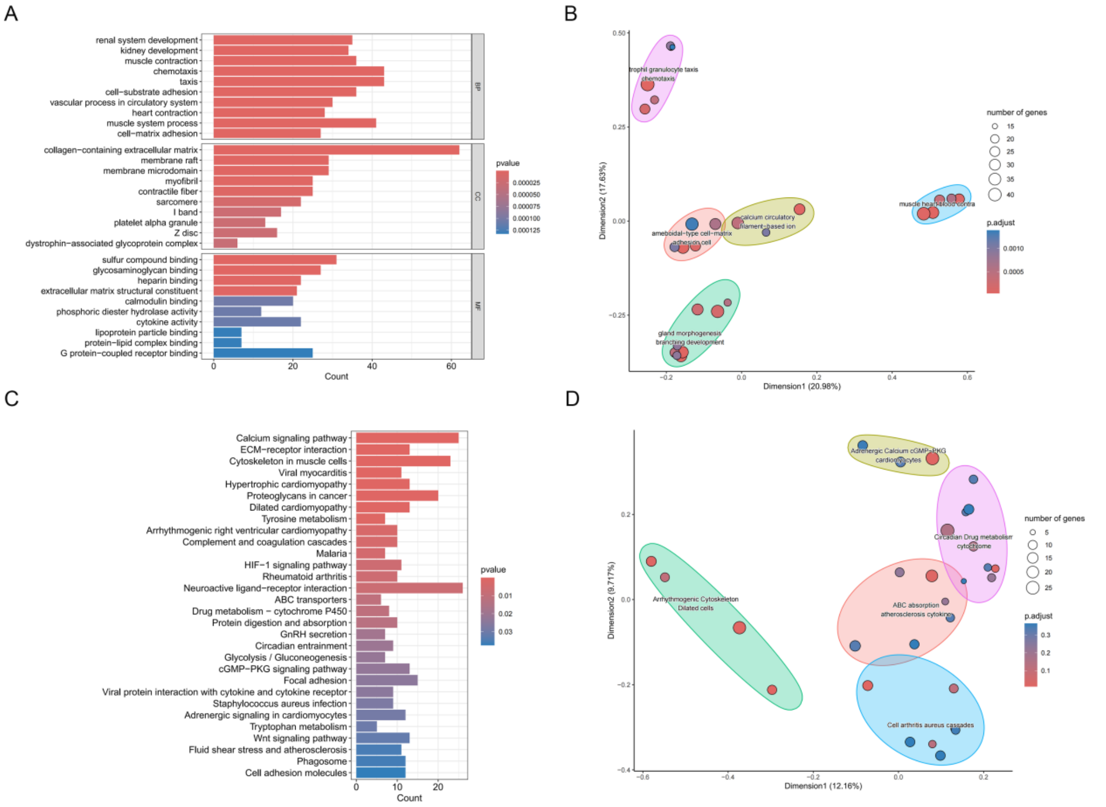

Supplementary Figure S3. Functional Synergy and Pathway Dysregulation in Lung Cancer DEGs. A. GO Biological Processes: Bar plot of enriched terms ranked by gene count and *P*-value (top term: renal system development). B. GO Scatterplot: Dimension-reduced visualization with elliptical clusters highlighting functionally related terms (color: adjusted *P*-value). C. KEGG Pathways: Bar plot of enriched pathways ranked by significance. D. KEGG Scatterplot: Elliptical clusters of pathways grouped by functional similarity (bubble size: gene count).

LASSO Regression Prioritizes SLC16A7, DDAH2, and WDR38 as Core Lung Cancer Biomarkers

LASSO regression identified three genes (SLC16A7, DDAH2, WDR38) with non-zero coefficients at the optimal λ (0.2626) (Supplementary Figure S4). The cross-validation curve (Supplementary Figure S4A) demonstrated minimal binomial deviance (y-axis: 0.4–0.8) across decreasing log(λ) values (x-axis: -5 to -1), with a sharp increase in deviance beyond λ = 0.2626 (red dashed line), confirming this threshold as optimal. The coefficient path plot (Supplementary Figure S4B) revealed progressive shrinkage of gene coefficients as λ increased, with only SLC16A7, DDAH2, and WDR38 retaining non-zero coefficients (y-axis range: -0.5 to 1.0) at the selected λ. Feature importance ranking (Supplementary Figure S4C) positioned SLC16A7 as the top contributor (absolute coefficient = 4.0), followed by DDAH2 (3.5) and WDR38 (1.2). These results nominate these genes as critical drivers of lung cancer pathogenesis, warranting functional validation.

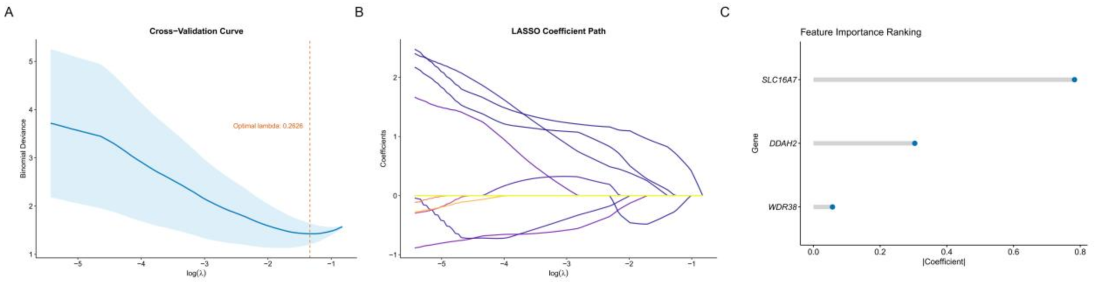

Supplementary Figure S4. LASSO-Driven Prioritization of Lung Cancer Biomarkers. A. Cross-Validation Curve: Relationship between log(λ) and binomial deviance (optimal λ = 0.2626 marked by dashed line). B. LASSO Coefficient Paths: Trajectories of gene coefficients across λ values (final selected genes highlighted). C. Feature Importance Ranking: Top three genes ranked by absolute LASSO coefficients (SLC16A7: highest).

Random Forest Model Identifies 30 Mechanistically Relevant Genes in Lung Cancer

The random forest model achieved stable classification (OOB error rate ≤0.06) after 250 trees, with error rates plateauing beyond this threshold (Supplementary Figure S5A). The error rate trajectory (orange dashed line) confirmed robust model performance below the 0.1 threshold. Feature importance analysis (Supplementary Figure S5B) prioritized 30 genes (Gini index: 0.04–0.12), including top-ranked WDR38 (0.12) and EFNB3 (0.10). High-scoring genes encompassed ion transporters (SLC16A7, CLEC5A), signaling regulators (FZD8, SEMA3D, JAKMIP2), and extracellular matrix modulators (WISP2, RETNLB). Horizontal bars displayed a viridis color gradient (purple: low importance, yellow: high), with gene labels ordered by descending Gini scores. These results nominate 30 genes as central to lung cancer pathogenesis, with implications for therapeutic targeting.

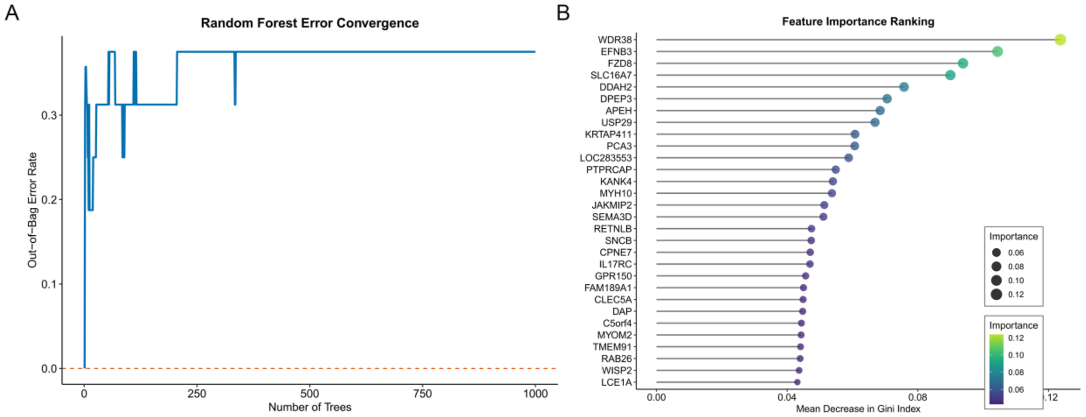

Supplementary Figure S5. Stability and Mechanistic Insights from Lung Cancer DEG Random Forest Analysis. A. Error Rate Convergence: Line plot of OOB error rate (y-axis) versus number of trees (x-axis). Orange dashed line marks the 0.1 error threshold. B. Feature Importance Ranking: Horizontal bar plot of 30 genes ranked by mean Gini index decrease (color gradient: viridis scale; bar length: importance score).

Overlapping Biomarkers DDAH2, SLC16A7, and WDR38 Exhibit Recurrent Mutations in Lung Cancer

Venn analysis identified three overlapping genes (DDAH2, SLC16A7, WDR38) between LASSO and RF models (Supplementary Figure S6A). Mutation profiling revealed WDR38 mutations in 0.98% (5/508) of LUAD and 0.82% (4/485) of LUSC cases (Supplementary Figure S6B), predominantly missense mutations (blue circles) distributed across the protein. SLC16A7 (Supplementary Figure S6C) exhibited sparse missense mutations (e.g., p.N58L, p.S125I) in LUAD (1.2% mutation rate), with no LUSC variants detected. DDAH2 (Supplementary Figure S6D) displayed a nonsense mutation hotspot (p.W356*, green circle) in both LUAD (0.2%) and LUSC (0.6%), alongside missense variants. Gray backgrounds denote conserved protein domains (e.g., amidinotransferase domain in DDAH2). These results nominate DDAH2, SLC16A7, and WDR38 as recurrently mutated, functionally relevant biomarkers in lung cancer.

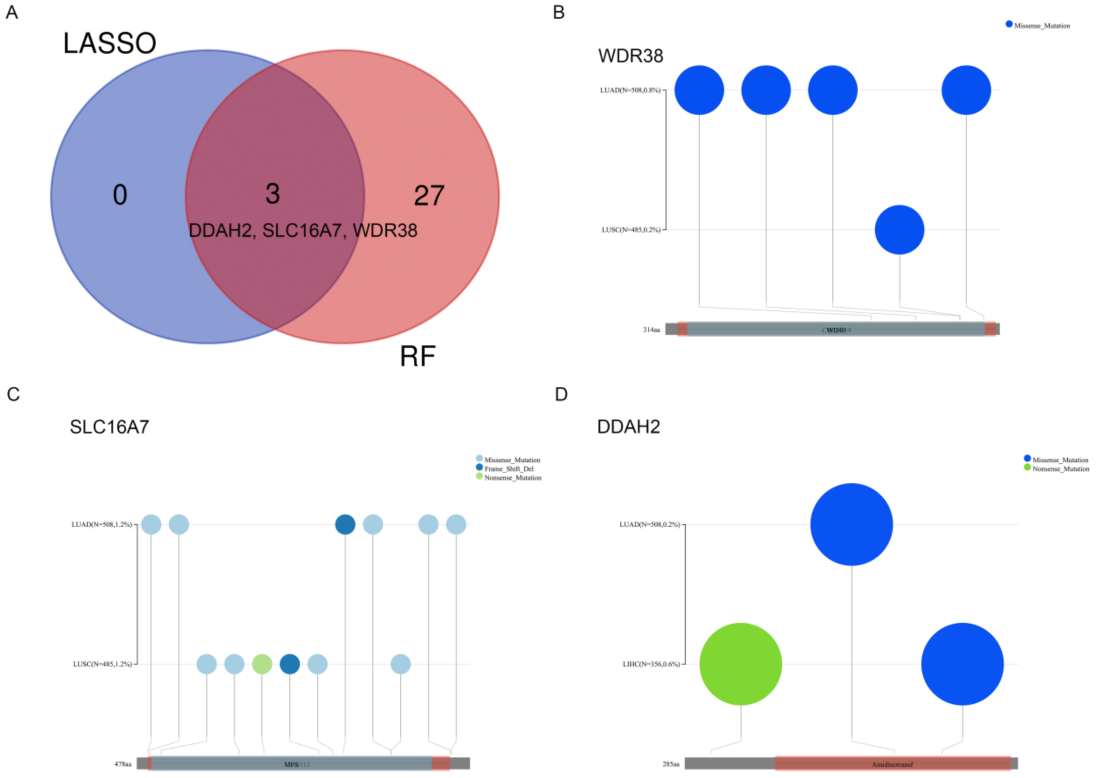

Supplementary Figure S6. Mutation Hotspots and Functional Domains of Prioritized Lung Cancer Biomarkers. A. Overlapping Genes: Venn diagram intersecting LASSO and RF-selected genes (3 overlaps). B. WDR38 Mutations: Mutation distribution (blue: missense; gray: protein domains; labels: residue positions). C. SLC16A7 Mutations: Missense variants (blue) mapped to transmembrane regions (gray domains). D. DDAH2 Mutations: Nonsense hotspot (green, p.W356*) and missense variants (blue) in amidinotransferase domain.
